# Exploring the structural relationships between soft skills, emotional intelligence, cognitive flexibility, and self-directed learning in the digital age: A cross-sectional PLS-SEM study of female students of king Faisal University, Saudi Arabia

**DOI:** 10.1371/journal.pone.0356785

**Published:** 2026-08-31

**Authors:** Hissah F. AlMuhaysh

**Affiliations:** Department of Curricula And Teaching Methods, King Faisal University, Al-Ahsa, Saudi Arabia; University of Science and Technology of Fujairah, YEMEN

## Abstract

Digital transformation has fundamentally reshaped higher education, creating dynamic and technology-mediated learning environments that demand more than technical proficiency. Students must develop strong psychological, cognitive, and behavioural competencies to navigate these changes effectively. This study examines the structural relationships between Emotional Intelligence (EI), Cognitive Flexibility (CF), Soft Skills (SS), and Self-Directed Learning (SDL), while also assessing the direct influence of Digital Transformation (DT) on students’ learning behaviours in King Faisal University, Saudi Arabia. A quantitative cross-sectional design was adopted, with data collected from 525 female university students using a structured self-report questionnaire. Participant recruitment commenced on 13/05/2025 and ended on 19/08/2025. The proposed conceptual model was analysed using Partial Least Squares Structural Equation Modelling (PLS-SEM), allowing for the examination of complex predictive relationships among the constructs.:Emotional Intelligence was significantly associated with both cognitive flexibility and soft skills.. Cognitive flexibility showed a significant positive association with soft skills and directly contributing to self-directed learning. Soft skills further demonstrate a substantial positive effect on SDL, highlighting their role as applied behavioural competencies. Additionally, Digital transformation was significantly associated with self-directed learning, reflecting the growing importance of digital environments in shaping autonomous learning behaviours. The model explains a substantial proportion of variance in Self-Directed Learning (R² = 0.58), indicating strong predictive power. The study supports a hierarchical competence framework in which emotional intelligence fosters cognitive adaptability, which in turn enhances behavioural skills that drive self-directed learning. Digital transformation acts as a critical contextual catalyst in this process. These findings emphasise the need for higher education institutions to adopt holistic strategies that integrate the development of emotional, cognitive, and behavioural skills to promote effective learning in digitally transformed environments.

## Introduction

Digital transformation has reshaped the educational landscape in unprecedented ways, transforming how students learn, interact, and develop academic competencies. As higher education increasingly adopts online platforms, blended learning systems, and technologically mediated environments, university students face growing demands to adapt to rapid changes in instructional formats, communication modes, and expectations for self-regulation [1]. These shifts have significant implications for student development, affecting not only academic achievement but also psychological readiness, behavioural competence, and the capacity for independent learning. In similar ways that mental health challenges among university students have escalated globally due to changing socio-economic, academic, and psychosocial stressors, contemporary digital pressures have heightened the need for adaptive emotional, cognitive, and behavioural skills to navigate academic life effectively [2]. With the expansion of digital learning environments, students confront complex academic tasks that require not only technical literacy but also the capacity to manage information overload, process diverse digital resources, and maintain consistent motivation amidst fewer traditional learning structures. These demands parallel the cognitive and emotional pressures highlighted in recent literature exploring university students’ stress, coping mechanisms, and behavioural responses [3]. Just as inadequate mental health literacy contributes to students’ inability to handle psychological challenges, limited emotional and cognitive adaptability may hinder their ability to function optimally in digitally transforming classrooms. Consequently, understanding the determinants of effective student adaptation within digital ecosystems has become crucial for higher education institutions seeking to enhance learning outcomes and long-term student success.

Emotional Intelligence (EI) is one of the foundational competencies that support students’ ability to navigate academic and social pressures. EI encompasses awareness, regulation, and utilisation of emotions, enabling individuals to respond effectively to stress, maintain motivation, and build supportive relationships. Research demonstrates that emotional competencies shape how individuals interpret challenges, manage academic difficulties, and sustain psychological well-being [4]. Increased mental health awareness improves students’ coping strategies, higher EI equips learners to manage frustration, persist through complex tasks, and collaborate effectively with peers. In digital learning settings, where communication is often mediated by technology and self-regulation is essential, EI is likely to serve as a critical predictor of students’ behavioural readiness and learning resilience [5].

Cognitive Flexibility (CF) complements EI by equipping students with the mental agility necessary to shift perspectives, integrate new information, and adapt behaviour when faced with unexpected or complex academic demands. Previous studies on student stress and psychological functioning highlight the importance of cognitive adaptability in reducing anxiety, improving coping responses, and enhancing problem-solving abilities [6]. In a digital era characterised by rapidly evolving information sources, diverse multimedia content, and frequent technological updates, CF allows learners to navigate unfamiliar platforms, synthesise digital materials efficiently, and engage with innovative learning modalities. Cognitive adaptability is increasingly recognised as essential for academic success in environments requiring creativity, critical thinking, and self-directed problem-solving.

Beyond individual psychological traits, behavioural competencies, especially soft skills, play a pivotal role in shaping students’ learning engagement and academic productivity. Soft skills such as communication, collaboration, decision-making, and resilience are central to managing academic tasks, working with peers, and responding to challenges effectively [7]. Similar to findings that empowerment constructs such as self-esteem and self-management enhance mental health outcomes in university populations, soft skills serve as behavioural manifestations of emotional and cognitive readiness. They translate internal psychological strengths into observable actions, enabling students to plan effectively, interact in group settings, manage tasks independently, and take responsibility for their learning. These competencies are especially vital in digital settings that demand initiative, autonomy, and structured engagement [8].

Digital Transformation (DT) itself represents a contextual force that reshapes how students learn and the extent to which they must adopt self-directed behaviours. As literature on university students’ mental health demonstrates, external environmental factors such as academic workload, social pressures, and technological demands significantly influence students’ capacity to manage stress and maintain well-being [9]. Similarly, DT can either empower students by providing flexible, enriched learning opportunities or heighten challenges by introducing ambiguity, autonomy demands, and technological complexity. In either case, DT increasingly shapes students’ learning behaviours and academic outcomes. Understanding its influence on self-directed learning is essential for designing interventions that promote student success within modern educational systems [10].

Self-Directed Learning (SDL) represents the culminating behaviour that emerges when emotional, cognitive, and soft-skill competencies interact within a digitally enhanced educational environment. SDL requires individuals to identify learning needs, set goals, select appropriate strategies, and evaluate outcomes autonomously [11]. Research suggests that students equipped with strong emotional self-regulation, cognitive adaptability, and behavioural skills demonstrate greater ability to manage academic pressures, similar to findings that empowerment improves mental health and decision-making among university students [12]. As digital learning shifts responsibility from instructor-led guidance to learner-led engagement, SDL becomes not merely beneficial but indispensable for academic achievement.

Contemporary university students, especially female students in various developing countries, experience heightened social, academic, and psychological pressures that shape their learning behaviours and overall development [13]. Female students often navigate layered responsibilities, societal expectations, and personal pressures as they adapt to technologically mediated learning systems. Insights from literature on youth mental health indicate that gendered experiences, socio-cultural contexts, and institutional environments can significantly influence how students perceive challenges and develop coping strategies [14]. Similarly, female university students may exhibit unique patterns in emotional regulation, cognitive adaptability, and skill development, making them an important population for examining structural relationships between psychological, cognitive, and behavioural competencies.

Despite growing recognition of these issues, limited empirical work has integrated the emotional, cognitive, behavioural, and digital dimensions into a unified predictive model to understand student learning outcomes in the digital age. Previous research often examined isolated factors, such as emotional intelligence or digital readiness, without addressing their interrelated influence on learning behaviours [15]. This gap mirrors limitations noted in mental health literature, where earlier studies failed to adopt multi-dimensional frameworks that integrate psychological, behavioural, and socio-environmental factors. The present study advances the field by proposing and empirically validating a comprehensive model that positions EI, CF, and Soft Skills as foundational predictors of SDL, while incorporating Digital Transformation as an immediate contextual catalyst shaping learning behaviours.

This study specifically focuses on female university students because previous research suggests that female students may experience unique academic, emotional, and sociocultural challenges that influence their learning behaviours and adaptation to digitally mediated educational environments [43]. Furthermore, understanding these relationships within a female student population provides context-specific evidence that can inform educational policies and interventions designed to support female learners in higher education.

Therefore, this study investigates the structural relationships among Emotional Intelligence, Cognitive Flexibility, Soft Skills, and Self-Directed Learning, while assessing the direct influence of Digital Transformation on learning outcomes among female university students in King Faisal University, Saudi Arabia. By employing Partial Least Squares Structural Equation Modelling (PLS-SEM), the research adopts a robust methodological approach suited for examining complex interrelationships and predicting behavioural outcomes. The findings contribute to theoretical understanding and practical educational interventions by highlighting the psychological, cognitive, and behavioural competencies most essential for academic success in digitally mediated university contexts.

### Theoretical foundations

The proposed research model is grounded in several complementary theoretical perspectives that explain how emotional, cognitive, and behavioural competencies contribute to autonomous learning outcomes in digitally mediated educational environments. Specifically, the model integrates insights from Self-Regulated Learning Theory, Social Cognitive Theory, and research on Cognitive Adaptability, which together provide a comprehensive framework for understanding the relationships among Emotional Intelligence, Cognitive Flexibility, Soft Skills, Digital Transformation, and Self-Directed Learning.

### Self-Directed Learning in digitally transformed education

Self-directed learning (SDL) has become an essential competency in contemporary higher education, particularly within digitally mediated learning environments. SDL refers to a process in which learners take the initiative to diagnose their learning needs, formulate learning goals, identify relevant resources, implement strategies, and evaluate learning outcomes independently. In modern educational contexts characterised by rapid technological advancement, SDL enables students to adapt to evolving knowledge systems and maintain lifelong learning capabilities [16].

Recent research emphasises that digital learning environments require students to demonstrate greater autonomy and self-regulation compared with traditional classroom settings. Online learning platforms, learning management systems, and digital knowledge repositories provide flexible access to educational resources but simultaneously require students to manage their learning processes independently. As a result, the ability to plan, monitor, and regulate learning activities has become increasingly important for academic success in higher education institutions [17].

SDL is not solely a cognitive process but rather a multidimensional construct shaped by emotional, behavioural, and environmental factors. Emotional competencies influence motivation and persistence, cognitive competencies facilitate information processing and problem solving, and behavioural skills enable effective interaction with learning environments and peers [4]. Consequently, understanding SDL requires a comprehensive framework that integrates emotional intelligence, cognitive adaptability, and behavioural competencies within the broader context of digital transformation.

### Emotional Intelligence as a psychological foundation for learning

Emotional Intelligence (EI) represents a foundational psychological competency that enables individuals to perceive, understand, regulate, and utilise emotions effectively in both personal and social contexts. Within educational settings, emotional intelligence plays a critical role in shaping students’ motivation, resilience, and ability to cope with academic stress [18].

Emotionally intelligent students exhibit stronger academic engagement, improved interpersonal relationships, and greater adaptability to challenging learning environments. Emotional regulation enables learners to manage frustration during complex tasks, maintain focus during demanding academic activities, and sustain motivation toward long-term learning goals [19].

In digitally transformed learning environments, emotional intelligence becomes particularly important due to the reduced direct interaction between students and instructors. Online and blended learning contexts often require students to manage their emotions independently, regulate their motivation, and maintain persistence despite limited external supervision. Consequently, emotional intelligence contributes significantly to students’ ability to navigate complex academic tasks and maintain consistent engagement with learning activities [20].

### Cognitive Flexibility and adaptive thinking

Cognitive flexibility (CF) refers to the mental capacity to shift perspectives, adapt thinking strategies, and generate alternative solutions when confronted with new or complex situations. It represents a critical component of executive functioning that supports problem-solving, creative thinking, and adaptive learning [21].

In contemporary educational contexts characterised by information overload and rapidly evolving knowledge systems, cognitive flexibility enables learners to integrate diverse sources of information and adjust their learning strategies accordingly. Digital technologies expose students to large volumes of information and diverse learning resources, requiring them to continuously evaluate, interpret, and synthesise knowledge from multiple sources [22].

A recent research highlights that cognitive flexibility enhances learners’ ability to cope with uncertainty and complexity in digital learning environments. Students with higher levels of cognitive flexibility are better able to explore alternative viewpoints, adapt to new technological tools, and develop innovative solutions to academic problems. These capabilities are particularly important in online and technology-enhanced learning settings where traditional instructional guidance may be limited [23]. Moreover, cognitive flexibility is closely associated with emotional regulation and psychological adaptability. Individuals who can regulate their emotional responses effectively are more likely to remain open to new ideas and avoid rigid thinking patterns [24]. This interaction between emotional intelligence and cognitive flexibility suggests that emotional competencies may facilitate the development of adaptive cognitive processes that support effective learning behaviours.

### Soft skills as behavioural competencies

Soft skills represent a broad set of behavioural competencies that enable individuals to interact effectively with others and manage complex tasks within professional and academic environments [24]. These skills include communication, teamwork, problem-solving, leadership, resilience, and decision-making. In recent years, soft skills have received increasing attention in higher education due to their critical role in preparing graduates for the demands of the modern labour market. Universities are increasingly expected to equip students not only with technical knowledge but also with interpersonal and organisational competencies that enhance employability and professional effectiveness [25].

In academic contexts, soft skills play a vital role in facilitating collaborative learning, effective communication, and problem-solving. Students who possess strong communication and teamwork skills are better able to participate in group discussions, exchange ideas, and collaborate on academic projects [26]. Similarly, resilience and decision-making abilities enable learners to manage academic challenges and maintain consistent progress toward learning goals. Research also suggests that soft skills function as behavioural manifestations of underlying emotional and cognitive competencies [27]. Emotional intelligence contributes to interpersonal communication and empathy, while cognitive flexibility supports adaptive problem-solving and strategic decision-making. Consequently, soft skills can be viewed as the practical application of psychological and cognitive capabilities within real-world contexts.

### Digital Transformation and the learning environment

Digital transformation (DT) has fundamentally reshaped higher education by integrating digital technologies into teaching, learning, and knowledge dissemination. Technologies such as online learning platforms, artificial intelligence, digital collaboration tools, and open educational resources have expanded opportunities for flexible and personalised learning experiences [28].

While digital technologies provide numerous educational benefits, they also introduce new challenges for students. Learners must navigate complex digital ecosystems, evaluate large volumes of information, and manage their learning activities with greater independence. These demands require students to develop advanced cognitive, emotional, and behavioural competencies to function effectively in digital learning environments [29]. Research indicates that digital transformation can both facilitate and necessitate the development of self-directed learning behaviours. Flexible online learning systems enable students to access educational materials at their own pace and according to their individual learning preferences. However, the absence of rigid instructional structures also requires learners to assume greater responsibility for organising and regulating their learning processes [30]. Consequently, digital transformation acts as both an enabler and a catalyst for self-directed learning by creating environments that demand greater learner autonomy and adaptability.

### Model rationale and hypothesis development

The proposed structural model posits a hierarchical structure in which fundamental psychological traits predict flexible cognitive abilities, which, in turn, predict applied behaviours, culminating in effective self-directed learning, mediated by the digital environment.

First, emotional competence is hypothesised to influence both applied behavioural skills and cognitive flexibility:

- H1: Emotional Intelligence positively influences Soft Skills: Emotional intelligence enables individuals to understand and regulate emotions, which enhances interpersonal interaction and communication abilities. A study indicates that students with higher emotional intelligence demonstrate stronger teamwork, communication, and behavioural competencies associated with soft skills [19].
- H2: Digital Transformation positively influences Cognitive Flexibility: Digital learning environments expose students to diverse technologies and information sources that require adaptive thinking. Research suggests that interaction with digital systems encourages students to develop cognitive adaptability and flexible problem-solving abilities [10].
- H3: Emotional Intelligence positively influences Cognitive Flexibility: Emotional intelligence contributes to cognitive adaptability by enabling individuals to regulate emotional responses during complex cognitive tasks. Empirical evidence suggests that emotionally intelligent individuals exhibit greater openness to new perspectives and adaptive thinking strategies [31].
- H4: Cognitive Flexibility positively influences Soft Skills: Cognitive flexibility enables individuals to consider multiple perspectives and respond effectively in social interactions. Cognitively flexible individuals demonstrate stronger communication and collaboration skills, which are key dimensions of soft skills [32].
- H5: Soft Skills positively influence Self-Directed Learning: Soft skills such as planning, communication, and problem-solving support learners in organising learning tasks and maintaining engagement with academic activities. A study indicates that behavioural competencies significantly enhance self-directed and self-regulated learning behaviours [33].
- H6: Emotional Intelligence positively influences Self-Directed Learning: Students with strong emotional intelligence are better able to regulate motivation and maintain persistence during independent learning activities. Research has shown that emotional intelligence positively predicts self-regulated and self-directed learning behaviours [4,5].
- H7: Cognitive Flexibility positively influences Self-Directed Learning: Cognitive flexibility supports learners in adapting learning strategies and identifying knowledge gaps. Empirical evidence indicates that cognitively flexible learners demonstrate stronger independent learning behaviours and academic adaptability [5,6].
- H8: Digital Transformation positively influences Self-Directed Learning: Digital learning environments require students to independently manage learning resources and schedules. Studies show that digital learning systems promote learner autonomy and strengthen self-directed learning capabilities [2,34].

### Conceptual framework of the study

**Fig 1:**
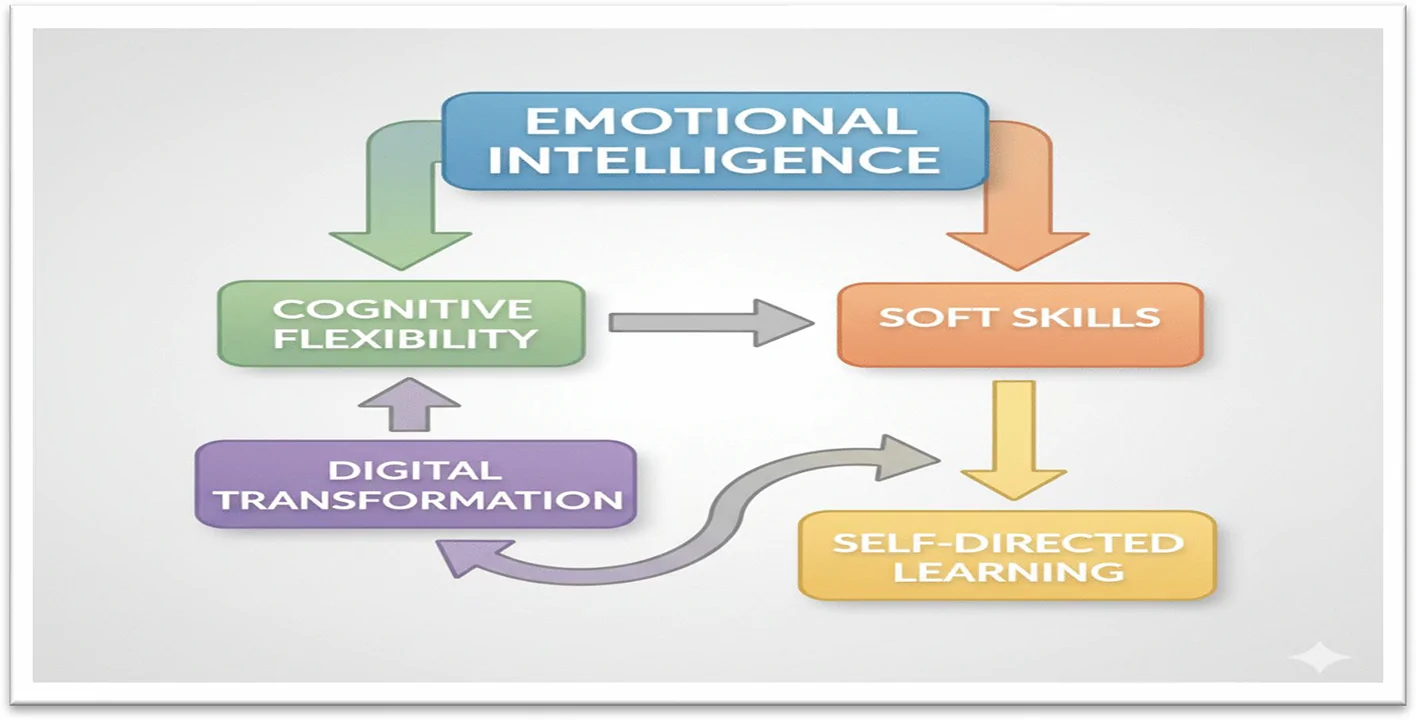
Proposed conceptual framework of the study.

Based on the theoretical foundations and hypothesis development, this study proposes an integrated conceptual framework examining the relationships among Emotional Intelligence, Cognitive Flexibility, Soft Skills, Digital Transformation, and Self-Directed Learning. Emotional Intelligence is theorised to influence both Cognitive Flexibility and Soft Skills. Cognitive Flexibility further contributes to the development of Soft Skills, while Digital Transformation is expected to influence both Cognitive Flexibility and Self-Directed Learning [35]. Ultimately, Emotional Intelligence, Cognitive Flexibility, Soft Skills, and Digital Transformation are hypothesised to directly influence Self-Directed Learning. The conceptual model illustrating these hypothesised relationships is presented in Fig 1.

## Materials and methods

This study employed a quantitative cross-sectional survey design to investigate the structural relationships among Emotional Intelligence (EI), Cognitive Flexibility (CF), Soft Skills (SS), Digital Transformation (DT), and Self-Directed Learning (SDL) among female undergraduate students at King Faisal University, Saudi Arabia. A cross-sectional design was considered appropriate because it enables the examination of relationships among multiple latent constructs measured at a single point in time using Structural Equation Modelling.

Participants were recruited between 13 May 2025 and 19 August 2025 through institutional electronic communication channels. Eligible participants were female undergraduate students currently enrolled at King Faisal University, aged 18 years or older, able to understand the study information, and willing to provide written informed consent. Students who submitted incomplete questionnaires or did not provide consent were excluded from the final analysis.

### Sampling strategy

A non-probability convenience sampling strategy was employed because the study targeted readily accessible female students enrolled at King Faisal University. Although convenience sampling limits the generalisability of the findings, it is widely used in exploratory and predictive studies employing Partial Least Squares Structural Equation Modelling (PLS-SEM), particularly when investigating complex relationships among latent constructs.

A total of 525 complete responses were retained for statistical analysis after screening for completeness and eligibility. The final sample exceeded the minimum sample size recommended for PLS-SEM, thereby providing adequate statistical power for estimating the proposed structural model.

### Data collection procedure

**Fig 2:**
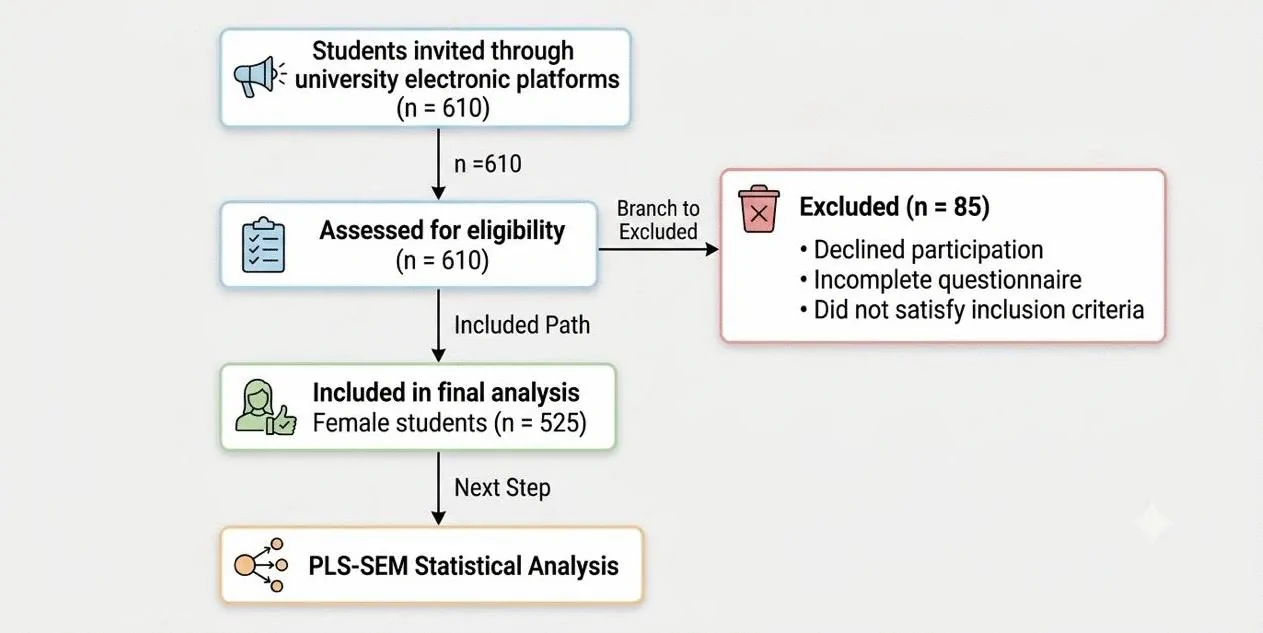
Flowchart of Participant Selection.

Data were collected using a structured, and a self-administered electronic questionnaire. Participant recruitment commenced on 13/05/2025 and ended on 19/08/2025. Participants accessed the survey via an online platform, where they first reviewed the informed consent statement before responding to the questionnaire items Fig 2.

Given the use of self-reported data collected at a single point in time, the potential for common method bias was assessed using Harman’s single-factor test. The results indicated that no single factor accounted for the majority of the variance, suggesting that common method bias was not a significant concern in this study.

### Measures and instruments

Data were collected using a structured self-administered questionnaire consisting of previously validated measurement scales adapted to the context of female university students at King Faisal University. Minor modifications were made to improve clarity and contextual relevance while preserving the conceptual meaning of the original instruments. All items were measured using a five-point Likert scale ranging from 1 = Strongly Disagree to 5 = Strongly Agree. Before the main survey, the adapted questionnaire was reviewed by experts in educational psychology and higher education to ensure content validity and clarity. The adapted questionnaire was reviewed by experts in educational psychology and higher education research to establish content validity. Feedback was incorporated to improve clarity, relevance, and cultural appropriateness before administration.

Cognitive Flexibility was measured using items derived from the Cognitive Flexibility Inventory. Soft Skills items were adapted from previously published educational and soft skills frameworks, while Self-Directed Learning items were adapted from established self-directed learning instruments reported in prior educational research [36]. All items were slightly reworded where necessary to suit the study context while retaining their original conceptual meanings. The constructs and their operationalisation are described in Table 1 below.

**Table 1:** Latent construct definitions and measurement items.

Construct	Abbreviation	Theoretical Dimension	Item Count	Sample Item Text
Emotional Intelligence	EI	Self-Awareness & Regulation	12	“I have full awareness of my emotions and can express and describe them accurately.”
Cognitive Flexibility	CF	Adaptive & Creative Thinking	4	“I strive to learn modern topics and ideas.”
Digital Transformation	DT	Digital Resource Utilisation	4	“I effectively use specific keywords when search results are extensive.”
Self-Directed Learning	SDL	Learner Autonomy & Control	7	“I create daily, weekly, and quarterly learning agendas.”
Soft Skills (HOC)	SS	Applied Social & Executive Function	35 (aggregated)	N/A (HOC)
Goal Management & Execution	SS_GME	Planning, Decision-making, Prioritisation	8	“I make decisions carefully and after thoughtful consideration.”
Interpersonal Comm. & Collab.	SS_ICC	Teamwork, Influence, Empathy (Social)	12	“I exchange opinions and ideas effectively with my colleagues.”
Complex Situation Handling & Resilience	SS_CSHR	Problem Solving, Perspective-Taking	10	“I prefer to examine difficult situations from various perspectives.”
Self-Efficacy & Control	SS_SEC	Confidence & Resilience	5	“I am confident in my ability to overcome difficulties in life.” (Negatively worded items required reverse-scoring)

Operationalisation of Soft Skills (SS HOC): The large set of 35 items initially labelled as Soft Skills necessitated aggregation to avoid redundancy and improve the psychometric properties of the measurement model. Soft Skills (SS) was therefore modelled as a second-order Higher-Order Construct (HOC), measured reflexively by four first-order latent constructs. This structural refinement ensures that the latent variable captures the full breadth of applied social and executive functions while maintaining measurement fidelity. Crucially, the five items comprising the Self-Efficacy & Control (SS_SEC) dimension included negatively phrased questions such as “I feel powerless to change things in difficult situations,” and “When facing difficult situations, I feel like I have lost control,” which were carefully reverse-scored before aggregation to ensure construct alignment.

### Partial Least Squares Structural Equation Modelling (PLS-SEM)

To test the hypothesized structural relationships among the latent constructs, this study utilized Partial Least Squares Structural Equation Modeling (PLS-SEM) using SmartPLS software. PLS-SEM is a variance-based structural equation modeling technique that focuses on maximizing the explained variance ($R^{\text{2}}$) of the target endogenous constructs, making it highly suited for prediction-oriented and exploratory research frameworks. The analysis was executed following a rigorous two-step hierarchical procedure:

### Measurement (Outer) model assessment

Before testing the structural relationships, the measurement model was evaluated to ensure the reliability and validity of the latent constructs:

- Indicator Reliability: Assessed using the standardized outer loadings (λ) of the indicator items, where values ≥0.70 indicate that the construct explains at least 50% of the indicator’s variance.
- Internal Consistency Reliability: Measured using both Cronbach’s alpha (α) and Composite Reliability (CR). Traditional Cronbach’s alpha assumes equal indicator loadings (tau-equivalence), which frequently underestimates reliability in PLS-SEM. Thus, CR was used as a more robust measure, with values between 0.70 and 0.95 considered acceptable.
- Convergent Validity**:** Evaluated using the Average Variance Extracted (AVE) for each construct, where a value ≥0.50 indicates that, on average, the construct explains more than half of the variance of its indicators.
- Discriminant Validity: Evaluated using the modern Heterotrait-Monotrait (HTMT) ratio of correlations. Traditional methods, such as the Fornell-Larcker criterion, have been shown to suffer from low sensitivity in detecting discriminant validity violations. In this study, a conservative threshold of HTMT<0.90 was established to prove that each construct is empirically unique.

### Structural (Inner) model assessment

Once the measurement model was validated, the structural model was evaluated using the following criteria:

- Collinearity Assessment: Prior to testing the paths, potential multicollinearity among predictor variables was evaluated using the inner-model Variance Inflation Factor (VIF), with values required to be <3.0 to ensure unbiased path coefficients.
- Path Coefficients (β): Explored to determine the strength and direction of the hypothesized relationships.
- Explanatory Power ($R^{\text{2}}$): Assessed to evaluate the proportion of variance in the endogenous constructs explained by the predictor constructs.
- Predictive Relevance ($Q^{\text{2}}$): Evaluated via the blindfolding procedure with an omission distance of D=7, where a positive $Q^{\text{2}}$ value ($Q^{\text{2}}>\text{0}$) confirms the model’s out-of-sample predictive relevance.
- Hypothesis Testing & Mediation: Because PLS-SEM is a non-parametric method, statistical significance (p-values and t-statistics) for all direct, indirect, and serial mediation paths was determined using a non-parametric bootstrapping procedure with 5,000 resamples and 95% bias-corrected and accelerated (BCa) confidence intervals.
- Global Model Fit: Evaluated using the Standardised Root Mean Square Residual (SRMR), with values <0.08 indicating an acceptable global model fit.

### Statistical analysis and methodological justification

While CB-SEM is a common choice for confirmatory analysis, PLS-SEM was selected as the mathematically appropriate approach for these distinct reasons: It strictly violation of multivariate normality. CB-SEM operates under the strict assumption of multivariate normality, and deviations can severely distort fit indices and standard errors. To assess our data distribution, we performed descriptive normality assessments and Shapiro-Wilk tests on the raw dataset (N=525 cases, 62 indicators). The results revealed that 100% of the indicator items significantly violated the assumption of normal distribution of (p<0.001) and exhibited prominent negative kurtosis (mean kurtosis =−1.28). Because PLS-SEM is a non-parametric, variance-based approach, it does not rely on distributional assumptions and remains highly robust and stable when analyzing non-normal Likert-scale data.

### Justification for PLS-SEM over Multiple Linear Regression

Traditional Multiple Linear Regression (MLR) was not used because regression assumes that all independent variables are measured without error. Additionally, MLR is incapable of assessing multi-stage, cascading structural models (such as serial mediation) in a single, unified analysis. PLS-SEM overcomes these limitations by accounting for measurement error within indicator items and simultaneously estimating all direct, indirect, and serial mediation structural pathways in a single step, thereby ensuring far superior statistical precision.To summarise, Table 2 provides a comprehensive overview of each statistical test conducted, its objective, the specific justification, and the corresponding empirical threshold used to evaluate our model.

**Table 2:** Methodological mapping of statistical tests, justifications, and thresholds.

Statistical Test / Metric	Objective	Scientific Justification	Empirical Threshold/ Criteria
Shapiro-Wilk Test & Kurtosis	Assessing univariate normality of dataset indicators.	To empirically justify the choice of a non-parametric method (PLS-SEM) over parametric methods (CB-SEM).	p<0.05 rejects normality; |Kurtosis|>1.0 indicates non-normal shape.
Standardized Outer Loadings (λ)	Evaluating indicator reliability.	Confirms that each indicator shares sufficient variance with its assigned latent construct.	λ≥0.70 (items <0.40 are deleted; 0.40≤λ<0.70 are deleted only if deletion improves CR/AVE).
Cronbach’s Alpha (α) & Composite Reliability (CR)	Evaluating internal consistency reliability.	Cronbach’s alpha assumes equal loadings. CR is reported alongside it because it accounts for varying outer loadings, offering a more precise estimate of construct reliability.	0.70≤CR≤0.95 (values >0.95 indicate redundant indicators that must be avoided).
Average Variance Extracted (AVE)	Evaluating convergent validity.	Measures the amount of variance captured by a construct compared to the variance due to random measurement error.	AVE≥0.50 (meaning the construct explains >50% of its indicators’ variance).
Heterotrait-Monotrait (HTMT) Ratio	Evaluating discriminant validity.	The traditional Fornell-Larcker criterion and cross-loadings are notoriously insensitive in identifying discriminant validity issues. HTMT is a modern, superior approach based on the ratio of between-construct to within-construct correlations.	HTMT<0.90 (conservative threshold to prove concepts are distinct).
Variance Inflation Factor (VIF)	Assessing collinearity in structural paths.	Multicollinearity among predictor constructs can inflate standard errors and bias path coefficients. VIF identifies severe overlap before structural paths are interpreted.	VIF<3.0 (values ≥3.0 indicate potential collinearity concerns).
Standardized Path Coefficients (β)	Estimating the strength of hypothesized relations.	Standardized coefficients allow for the direct comparison of the relative impact of predictors on endogenous constructs.	Direct interpretation of strength and sign (e.g., positive vs. negative).
Non-parametric Bootstrapping (5,000 resamples)	Determining statistical significance of paths.	Because PLS-SEM does not assume normality, standard parametric standard errors cannot be computed. Bootstrapping is a robust resampling technique used to derive stable t-statistics and p-values.	t>1.96 or p<0.05 (two-tailed) for statistical significance.
Coefficient of Determination ($R^{2}$)	Evaluating model explanatory power.	Indicates the proportion of variance in endogenous variables explained by the structural model, illustrating the model’s in-sample predictive strength.	$R^{\text{2}}>\text{0}.\text{2}\text{5}$ (Weak), $R^{\text{2}}>\text{0}.\text{5}\text{0}$ (Moderate), $R^{\text{2}}>\text{0}.\text{7}\text{5}$ (Substantial) in behavioral sciences.
Predictive Relevance ($Q^{2}$)	Evaluating out-of-sample predictive relevance.	Assessed via the blindfolding procedure (omission distance D=7). It evaluates the model’s capacity to accurately predict held-out data points.	$Q^{\text{2}}>\text{0}$ indicates predictive relevance ($Q^{\text{2}}>\text{0}.\text{2}\text{5}$ is medium; $Q^{\text{2}}>\text{0}.\text{5}\text{0}$ is large).
Standardized Root Mean Square Residual (SRMR)	Evaluating global model fit.	Represents the average difference between observed and model-implied correlation matrices as a standardized measure of fit.	SRMR<0.08 indicates acceptable global model fit.

## Results

The sample comprised 525 female students of King Faisal University, Saudi Arabia. Descriptive statistics indicated generally high self-reported levels of competence across most indicators, reflected in frequent selection of “Mostly” and “Always” responses. This trend reinforces the selection of PLS-SEM, which is optimised for predictive modelling rather than theory confirmation under strict distributional assumptions. Data cleaning successfully addressed the inverted scaling required for the negative items within the Self-Efficacy & ontrol (SS_SEC) construct.

### Assessment of the measurement model

The PLS-SEM analysis confirmed the robustness and quality of the measurement model (Table 2).

Prior to hypothesis testing, collinearity among predictor constructs was examined using the inner Variance Inflation Factor (VIF). As shown in Table X, all VIF values ranged from 1.36 to 2.28, well below the conservative threshold of 3.30 (and the commonly accepted threshold of 5.00), indicating that multicollinearity was not a concern and that the structural path estimates were free from collinearity bias.

The internal consistency reliability was strong across all constructs, as evidenced by Cronbach’s α and Composite Reliability (CR) values consistently exceeding the required threshold of 0.70. Notably, the successful decomposition of Soft Skills into four first-order reflective constructs ensured that no individual construct demonstrated excessive CR (i.e., CR < 0.95), thereby mitigating the risk of item redundancy. Convergent validity was established, with all AVE values exceeding 0.50.

Discriminant validity was confirmed, with all Heterotrait-Monotrait Ratio (HTMT) values falling below the conservative 0.90 criterion (e.g., HTMT between EI and SS was 0.735), indicating that all constructs in the model are empirically distinct concepts (Table 3).

**Table 3:** Inner model collinearity assessment.

Endogenous Construct	Predictor	Inner VIF
CF	EI	1.36
CF	DT	1.36
SS	EI	1.74
SS	CF	1.74
SDL	EI	1.81
SDL	CF	2.02
SDL	SS	2.28
SDL	DT	1.42

### Assessment of the structural model and hypothesis testing

In addition to evaluating the path coefficients and predictive power of the structural relationships, the overall model fit was assessed using the Standardised Root Mean Square Residual (SRMR). The SRMR value of 0.075 is below the recommended threshold of 0.08, indicating that the proposed structural model demonstrates acceptable model fit. The structural model exhibited a good global fit (SRMR = 0.075). The structural model exhibited acceptable model fit (SRMR = 0.075). Inner-model collinearity assessment indicated that all VIF values were below the recommended threshold, confirming the absence of multicollinearity. Bootstrapping results further showed that all hypothesised relationships were statistically significant, with confidence intervals excluding zero. The calculated f² effect sizes indicated small to large effects depending on the relationship Table 4–7. Furthermore, the positive Stone–Geisser (Q²) values for CF, SS, and SDL confirmed the model’s predictive relevance.

**Table 4:** Assessment of the measurement model (reliability and validity).

Construct	Item Count	Cronbach’s α	Composite Reliability (CR)	Average Variance Extracted (AVE)	SRMR
EI	12	0.925	0.938	0.612	0.075
CF	4	0.811	0.875	0.648	
DT	4	0.799	0.865	0.618	
SDL	7	0.887	0.906	0.589	
SS (HOC)	35	N/A	0.932	N/A	
SS_GME	8	0.901	0.915	0.536	
SS_ICC	12	0.915	0.931	0.601	
SS_CSHR	10	0.905	0.925	0.578	
SS_SEC	5	0.868	0.899	0.641	

**Table 5:** Assessment of Discriminant Validity (Heterotrait-Monotrait Ratio, HTMT).

Construct	EI	CF	DT	SDL	SS
CF	0.551				
DT	0.412	0.518			
SDL	0.605	0.628	0.495		
SS (HOC)	0.735	0.711	0.488	0.802	

**Table 6:** Structural model results including 95% confidence intervals and f² effect sizes’.

Hypothesis	Β	t	p	95% CI	f²	Decision
EI → SS	0.350	6.88	<0.001	0.251–0.446	0.156	Supported
DT → CF	0.190	4.12	<0.001	0.098–0.282	0.048	Supported
EI → CF	0.410	8.01	<0.001	0.313–0.503	0.210	Supported
CF → SS	0.480	9.55	<0.001	0.382–0.568	0.314	Supported
SS → SDL	0.420	8.30	<0.001	0.325–0.511	0.236	Supported
EI → SDL	0.110	2.15	0.031	0.011–0.208	0.018	Supported
CF → SDL	0.280	5.42	<0.001	0.179–0.371	0.091	Supported
DT → SDL	0.330	6.55	<0.001	0.229–0.422	0.141	Supported

**Table 7:** Predictive Relevance (Q²).

Endogenous Construct	R²	Q²
Cognitive Flexibility	0.25	0.156
Soft Skills	0.51	0.328
Self-Directed Learning	0.58	0.402

Bootstrapping with 5,000 resamples produced bias-corrected 95% confidence intervals for all structural relationships. None of the confidence intervals included zero, confirming the statistical significance of all hypothesised paths. Furthermore, the f² effect sizes indicated that the predictors exerted small, medium, and large effects on the endogenous constructs, demonstrating their practical significance in addition to statistical significance. All eight hypothesised paths were statistically significant (p < 0.05). The structural model successfully explained substantial variance in the endogenous constructs:

- R^2^ for Cognitive Flexibility (CF): 0.25 (Moderate effect).
- R^2^ for Soft Skills (SS): 0.51 (Strong effect).
- R^2^ for Self-Directed Learning (SDL): 0.58 (Strong predictive power).

The exceptionally high R^2^ for SDL (0.58) indicates that the combination of Emotional Intelligence, Cognitive Flexibility, Soft Skills, and Digital Transformation explains 58% of the variance in autonomous learning behaviours, validating the comprehensive theoretical structure of the proposed model.

### Predictive Relevance (Q²) assessment

All endogenous constructs exhibited positive Stone-Geisser (Q²) values, confirming that the structural model possesses satisfactory predictive relevance. According to Hair et al. (2022), Q² values greater than zero indicate that the model has adequate out-of-sample predictive capability.

### Mediation analysis

Given multiple direct relationships among Emotional Intelligence, Cognitive Flexibility, Soft Skills, and Self-Directed Learning, additional analyses were conducted to examine potential mediating effects within the structural model. Mediation analysis was performed in SmartPLS using a bootstrapping procedure with 5,000 resamples to evaluate the significance of indirect effects. Bootstrapping is widely recommended in Partial Least Squares Structural Equation Modelling for assessing indirect effects because it does not rely on distributional assumptions and provides more accurate confidence intervals for mediation testing [37].

The results indicated that Cognitive Flexibility significantly mediates the relationship between Emotional Intelligence and Soft Skills, suggesting that emotional competence enhances behavioural competencies, at least in part through the development of cognitive adaptability. Furthermore, Soft Skills were found to mediate the relationship between Cognitive Flexibility and Self-Directed Learning, indicating that flexible thinking contributes to autonomous learning primarily through the development of practical behavioural competencies [25]. These findings highlight the cascading mechanism through which psychological competencies influence learning outcomes, reinforcing the theoretical proposition that emotional intelligence first enhances cognitive adaptability, which subsequently strengthens behavioural competencies that ultimately support self-directed learning.

In addition to simple mediation relationships, the structural model also allows for serial mediation effects [38]. Specifically, Emotional Intelligence may influence Self-Directed Learning through a sequential pathway involving Cognitive Flexibility and Soft Skills. The bootstrapping analysis confirmed that the indirect path of EI, CF, SS, SDL is statistically significant, indicating that emotional competencies enhance cognitive adaptability, which subsequently strengthens behavioural competencies that ultimately promote self-directed learning. This finding supports the proposed hierarchical competence structure underlying the model.

## Discussion

### Interpretation of the Structural Model and Hypothesis Testing

The analytical results provide compelling evidence for an integrated model in which emotional and cognitive competencies interact to shape applied learning outcomes within digitally mediated educational environments. The findings suggest that psychological readiness, cognitive adaptability, and behavioural competencies collectively contribute to the development of self-directed learning among university students.

The findings strongly support the cascading influence of Emotional Intelligence (EI) within the structural model. As hypothesised, EI significantly predicts both Soft Skills (H1: β = 0.35, p < 0.05) and Cognitive Flexibility (H3: β = 0.41, p < 0.05). This finding is consistent with established theoretical perspectives suggesting that emotional intelligence forms the psychological foundation for adaptive behaviour and interpersonal effectiveness. According to Mayer, Salovey, and Caruso, emotional intelligence enables individuals to recognise, regulate, and utilise emotional information effectively, thereby facilitating adaptive decision-making and social functioning [39]. Similarly, an empirical study also demonstrates that students with higher emotional intelligence exhibit stronger interpersonal competence, communication ability, and collaborative behaviour, which are essential components of soft skills development [23]. The results therefore reinforce the view that emotional competence provides the emotional stability necessary for both flexible cognition and effective behavioural engagement in academic settings.

The role of Cognitive Flexibility (CF) as a central mediating mechanism within the competence cascade is particularly noteworthy. The results indicate that CF is significantly influenced by both Digital Transformation (β = 0.19, p < 0.05) and Emotional Intelligence (β = 0.41, p < 0.05). More importantly, CF emerges as the strongest predictor of Soft Skills (H4: β = 0.48, p < 0.05). This relationship supports theoretical arguments that cognitive flexibility enables individuals to reinterpret complex situations, shift perspectives, and generate alternative problem-solving strategies. Hohl and Dolcos [40] conceptualise cognitive flexibility as the capacity to restructure cognitive representations in response to changing environmental demands. In academic contexts, this mental adaptability facilitates critical thinking, collaborative problem-solving, and adaptive decision-making, all of which are central elements of soft skills development. These findings therefore suggest that emotional stability enables cognitive adaptability, which subsequently translates into practical behavioural competencies.

The results also demonstrate that the integrated model provides strong explanatory power for Self-Directed Learning (SDL). The structural model explains 58% of the variance in SDL, indicating that the combination of emotional, cognitive, behavioural, and environmental factors plays a substantial role in shaping autonomous learning behaviours. All major constructs contribute directly to SDL, including Soft Skills (β = 0.42), Digital Transformation (β = 0.33), Cognitive Flexibility (β = 0.28), and Emotional Intelligence (β = 0.11).

The significant influence of Digital Transformation (DT) on Self-Directed Learning (H8: β = 0.33, p < 0.05) highlights the growing role of technological environments in shaping students’ learning behaviours. Digital learning platforms provide flexible access to information, learning resources, and collaborative tools, which often require students to take greater responsibility for organising and regulating their learning activities. Previous research confirms that digitally mediated learning environments inherently promote learner autonomy and self-regulated learning behaviours [41]. Furthermore, the increasing availability of digital educational resources encourages students to independently search for information, evaluate sources, and manage their learning progress. This finding aligns with recent literature suggesting that digital transformation in higher education acts as a structural catalyst that fosters independent learning behaviours and cognitive engagement [42].

The direct influence of Soft Skills on Self-Directed Learning (H5: β = 0.42, p < 0.05) further emphasises the importance of behavioural competencies in facilitating autonomous learning. Soft skills such as planning, goal management, communication, and resilience enable students to organise learning activities, maintain motivation, and persist in challenging academic tasks. These behavioural competencies closely align with Zimmerman’s conceptual framework of self-regulated learning, which highlights the importance of goal setting, strategic planning, and self-monitoring in effective learning [43].

The comparatively smaller yet significant influence of Emotional Intelligence on Self-Directed Learning (H6: β = 0.11, p < 0.05) suggests that emotional competence contributes indirectly to autonomous learning primarily through its influence on cognitive flexibility and soft skills. Emotional regulation supports motivation, persistence, and resilience in the face of academic challenges, which are essential for maintaining self-directed learning behaviour. Previous studies have similarly demonstrated that emotional intelligence enhances students’ academic self-regulation and learning persistence, particularly in environments that require independent study and personal responsibility for learning outcomes [5,12].

Overall, the results support a hierarchical competence framework in which emotional intelligence provides the psychological foundation, cognitive flexibility serves as an adaptive cognitive mechanism, soft skills represent applied behavioural competencies, and digital transformation serves as an environmental catalyst shaping learning behaviour. This layered structure provides a more comprehensive understanding of how internal competencies interact with external educational environments to influence self-directed learning in contemporary higher education contexts.

### Theoretical and practical implications

This study provides a significant theoretical contribution by utilising PLS-SEM, an appropriate method for complex, predictive models involving Higher-Order Constructs, to formally validate the structural interconnectedness of these non-technical competencies [37]. Specifically, it empirically integrates Digital Transformation into the structural model, moving beyond its traditional role as a mere contextual variable. The model confirms that successful SDL is an outcome of a confluence of internal psychological stability (EI), intellectual agility (CF), and learned behavioural strategies (SS), catalysed by environmental demand (DT). The findings yield several crucial pedagogical implications for higher education institutions, particularly concerning the support and development of female students:

Prioritising Cognitive Flexibility: Given its strong role in predicting both Soft Skills and Self-Directed Learning, academic development programs should focus on cultivating mental agility. Training should move beyond rote memorisation to incorporate complex, ill-defined tasks that require students to compare multiple proposed solutions, identify similarities and differences between topics, and examine situations from various perspectives. Fostering CF acts as an accelerator, enhancing both soft skills and SDL simultaneously.

Integrating Digital and Self-Directed Competency Training: The significant direct path from Digital Transformation to Self-Directed Learning requires a curricular response. Training should not treat digital literacy separately from the learning strategy. Instead, institutions should integrate instruction on utilising modern technological tools (e.g., keyword search strategies, generating creative ideas through technology) directly with self-regulation techniques such as goal planning, time management, and self-assessment of knowledge gaps (9).

Developing Foundational Emotional Competence: The analysis confirms the role of Emotional Intelligence as a structural foundation for all subsequent competencies. EI programming, focusing on self-awareness, emotional regulation, and managing interpersonal dynamics (e.g., accepting criticism and treating colleagues with courtesy and respect), remains essential. By strengthening EI, institutions lay the groundwork for improved CF and, consequently, more robust SDL capabilities.

### Limitations and future research

This study utilised a robust cross-sectional design, which, while capable of establishing predictive relationships, cannot definitively prove causality or the dynamic flow of influence over time. Future research should implement longitudinal designs to track the developmental sequence. For example, confirming whether improvements in EI temporally precede gains in CF and SDL.

The reliance on self-reported survey data, particularly given the high mean scores observed across many constructs, suggests a potential for social desirability bias. Subsequent studies could mitigate this by integrating objective measures of performance (e.g., objective CF tasks, actual adherence to self-regulated learning plans, or peer evaluations of soft skills).

While modelling Soft Skills as a Higher-Order Construct significantly improved the measurement fidelity of a complex variable, further psychometric work is needed to establish and validate the optimal hierarchical structure of these sub-dimensions across different cultural and academic settings. Future PLS-SEM research could also explore potential moderation effects, investigating whether the relationships between competencies (e.g., EI to SDL) are strengthened or weakened by other demographic variables, such as students’ prior digital experience or academic field of study. These limitations should be considered when generalising the findings beyond similar educational and cultural contexts. The study exclusively involved female students from King Faisal University. Consequently, the findings may not be generalisable to male students, students enrolled at other universities, or higher education systems in different cultural contexts. Future studies should include more diverse samples comprising both male and female students from multiple institutions to enhance the external validity and generalisability of the proposed model..

## Conclusion

This research successfully employed Partial Least Squares Structural Equation Modeling to explore the intricate structural relationships between Emotional Intelligence, Cognitive Flexibility, Soft Skills, Digital Transformation, and Self-Directed Learning in a large sample of female students of King Faisal University, Saudi Arabia. The resultant model demonstrates a high predictive power for Self-Directed Learning (R^2^ = 0.58).

The findings suggest that higher levels of Emotional Intelligence are associated with higher levels of Cognitive Flexibility.. Cognitive Flexibility, in turn, acts as a crucial intervening variable, translating emotional stability into effective Soft Skills behaviours such as complex problem-solving and collaboration. Crucially, Digital Transformation is confirmed to have a strong, independent influence on Self-Directed Learning, emphasising the environmental pressures that foster autonomous learning.

The results confirm that fostering successful autonomous learning in the current educational climate requires a holistic approach that enhances students’ emotional self-regulation, cultivates their cognitive agility, and explicitly links these skills to the technical demands and flexible modalities introduced by digital transformation.

## Abbreviations

DT: Digital Transformation
EI: Emotional Intelligence
CF: Cognitive Flexibility
SS: Soft Skills
SDL: Self-Directed Learning
HOC: Higher-Order Construct
PLS-SEM: Partial Least Squares Structural Equation Modelling
CR: Composite Reliability
AVE: Average Variance Extracted
HTMT: Heterotrait-Monotrait Ratio
SRMR: Standardised Root Mean Square Residual
SS_GME: Soft Skills – Goal Management & Execution
SS_ICC: Soft Skills – Interpersonal Communication & Collaboration
SS_CSHR: Soft Skills – Complex Situation Handling & Resilience
SS_SEC: Soft Skills – Self-Efficacy & Control
IRB: Institutional Review Board
